# Accumulation of alpha-synuclein pathology in the liver exhibits post-translational modifications associated with Parkinson’s disease

**DOI:** 10.1016/j.isci.2024.111448

**Published:** 2024-11-23

**Authors:** Martin Hallbeck, Sara Ekmark-Lewén, Philipp J. Kahle, Martin Ingelsson, Juan F. Reyes

**Affiliations:** 1 Department of Biomedical and Clinical Sciences, Department of Clinical Pathology, Linköping University, Linköping, Sweden; 2 Department of Public Health and Caring Sciences, Molecular Geriatrics, Rudbeck Laboratory, Uppsala University, Uppsala, Sweden; 3 Laboratory of Functional Neurogenetics, Department of Neurodegeneration, Hertie Institute for Clinical Brain Research and German Center for Neurodegenerative Diseases, University of Tübingen, Tübingen, Germany; 4 The Krembil Brain Institute, University Health Network, Toronto, ON, Canada; 5 Tanz Centre for Research in Neurodegenerative Diseases, Departments of Medicine and Laboratory Medicine & Pathobiology, University of Toronto, Toronto, ON, Canada

**Keywords:** Biological sciences, Neuroscience, Molecular neuroscience

## Abstract

Accumulating evidence demonstrates that alpha-synuclein (α-syn) pathology associated with Parkinson’s disease (PD) is not limited to the brain, as it also appears in a select number of peripheral tissues including the liver. In this study, we identified a number of PD-associated α-syn post-translational modifications in the livers of (Thy-1)-h[A30P] mice, a mouse model of familial PD expressing human α-syn harboring the A30P mutation driven by a neuron-specific promoter. *Ex vivo*, we also demonstrate that human hepatocytes induce post-translational modifications following α-syn fibrillar (PFF) treatment. Moreover, such cells also degrade PFFs over time, whereas oligomeric assemblies are more resistant to degradation, but this process can be enhanced by autophagy stimulators. Collectively, our findings suggest that pathological α-syn is transported to the liver in a modified state or is modified upon arrival, which facilitates its clearance and detoxification, pointing to a role for the liver in the degradation of PD-associated pathology.

## Highlights

• Liver α-syn pathology in the A30P model of PD harbors PTMs associated with the PD brain
• Hepatocytes differentially cleave human α-syn assemblies and promote pS129 phosphorylation
• Pharmacological enhancers of autophagy promote human α-syn degradation in human hepatocytes

## Graphical abstract

**Figure:**
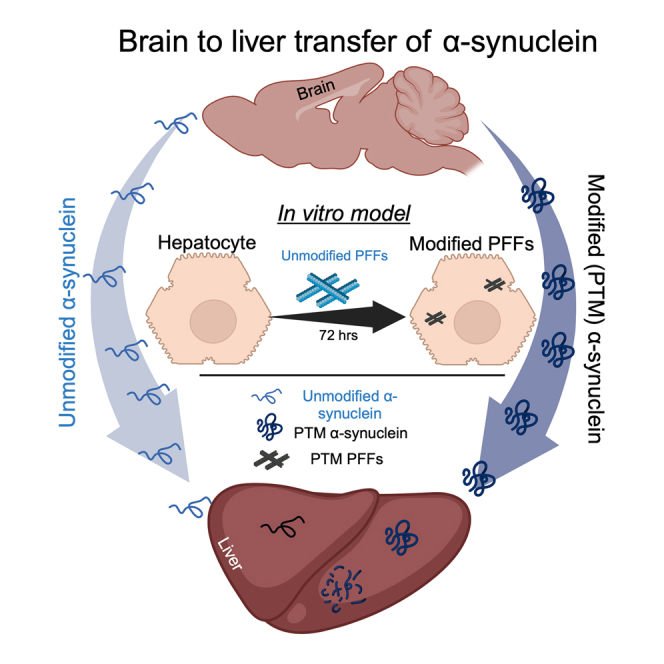

## Introduction

Parkinson’s disease (PD) is characterized by a progressive decline of motor and non-motor functions as well as a gradual deposition of alpha-synuclein (α-syn) proteins as Lewy bodies and Lewy neurites in the brain.^1^^,^^2^ In addition, point mutations (A30P, E46K, H50Q, G51D, A53 E/T) or multiplications of the α-syn gene (*SNCA*) have been shown to cause rare autosomal dominant forms of PD that either increase the levels of α-syn or promote the formation of toxic α-syn assemblies.^3^^,^^4^^,^^5^^,^^6^^,^^7^ Accumulating evidence now demonstrates that α-syn deposition is not limited to the brain as it also occurs within multiple organs outside the central nervous system (CNS).^8^^,^^9^ Indeed, α-syn pathology has been identified within the retina, skin, heart, gastrointestinal region, appendix, colon, and most recently within the liver^10^ (reviewed in^8^^,^^9^). Not surprisingly, α-syn pathology has also been identified in bodily fluids including blood, cerebrospinal fluid, saliva, and tear fluids.^11^^,^^12^^,^^13^^,^^14^^,^^15^^,^^16^ Collectively, these findings contribute to the ‘dual hit’ hypothesis in PD which suggests that pathology first affects either the olfactory bulb or the gut and then propagates to different regions of the brain as the disease progresses.^17^ This hypothesis implies that there are two PD subtypes, a brain-first and a body-first variant.^18^ Thus, α-syn pathology may spread either from the brain to the enteric or peripheral nervous system (PNS) or from the gut to the brain, explaining the interindividual differences in clinical manifestations during the disease progression.^18^

In recent years, the body-first hypothesis has received considerable attention. Mainly, a gut-to-brain spread of α-syn is strongly supported by early gastrointestinal symptoms which may appear years prior to neurodegeneration.^19^^,^^20^ Moreover, it has been suggested that appendix removal could reduce the PD risk and delay the age of onset,^21^ although a follow-up study did not show any such association.^22^ In rodent models of PD, multiple studies have shown that injection of α-syn in the mesenteric plexa of the gut leads to α-syn accumulation in the brain thus providing experimental evidence for this route being relevant for CNS pathology in PD.^23^ Accordingly, truncal vagotomy following α-syn injection was shown to prevent α-syn spread to the brain and subsequent neurodegeneration in the animals.^24^ Thus, current experimental evidence collectively strengthens the hypothesis that the accumulation and propagation of α-syn pathology from peripheral organs may be an early and critical feature of PD pathogenesis.

In previous work, we showed that human hepatocytes can take up α-syn assemblies via the gap junction protein connexin-32 (Cx32).^10^ Furthermore, we found an age-dependent accumulation of human α-syn pathology within the liver in multiple animal models of PD (L61, (Thy-1)-h[A30P])),^25^^,^^26^ and MSA (MBP29).^27^ Importantly, the accumulation of α-syn within the liver was not due to hepatic mRNA expression, indicating that α-syn deposits are derived directly from the brain or indirectly from other peripheral tissues.^10^ Moreover, we corroborated that α-syn pathology in neuropathologically confirmed PD cases can be found to a higher degree than in controls with no α-syn pathology in the brain.^10^ In the current report, we investigated the presence of α-syn post-translational modifications (PTMs) in the liver from aged (Thy-1)-h[A30P] mice (A30P).^10^ We now report the presence of hallmark PTMs associated with PD, including tyrosine nitration (nY39), phosphorylation (pY39, pS87 and pS129, Y133) and C-terminal truncation events (X-122).^28^^,^^29^^,^^30^^,^^31^ *Ex vivo*, we demonstrate that human hepatocytes (HuH-7) degrade pre-formed fibrils (PFF) more efficiently than oligomeric assemblies. However, by increasing autophagy using the pharmacological inhibitor rapamycin, we could enhance oligomeric α-syn degradation in a concentration-dependent manner. Moreover, *ex vivo* we also observe several PTMs that have been demonstrated *in vivo*. Taken together, our results demonstrate the presence of key pathological modifications associated with PD,^28^^,^^29^^,^^30^^,^^31^ also present in the liver of a mouse model of PD. Our findings suggest that α-syn aggregates are transported from the brain to the liver in a modified state or upon arrival, they undergo specific PTMs to facilitate their clearance and detoxification, suggesting a new role for the liver in the clearance of PD-associated pathology.

## Results

### Human α-syn aggregates in the (Thy-1)-h[A30P] liver are C-terminally modified

**Figure 1:**
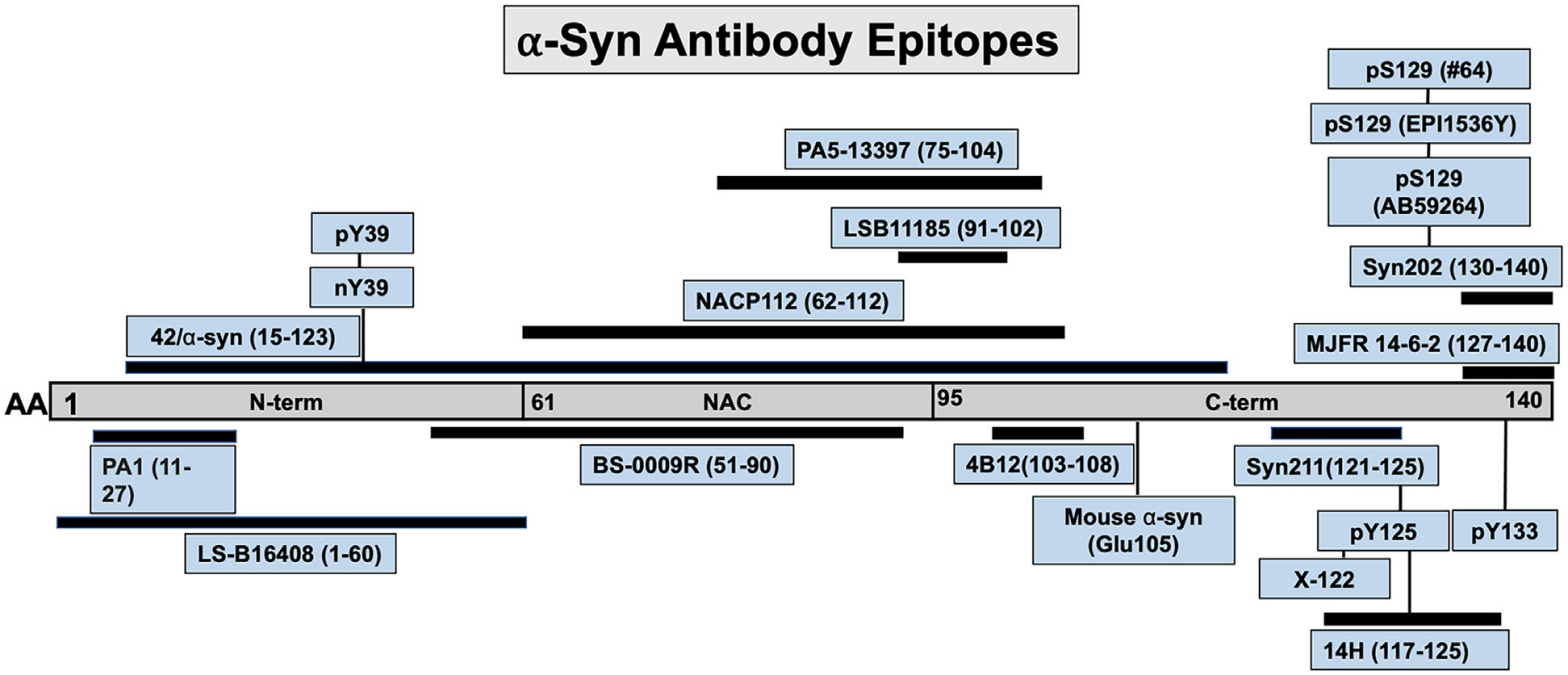
Human α-syn antibody diagram depicting all antibodies used in this study Each antibody epitope is represented schematically by lines under or above the α-syn protein.

**Figure 2:**
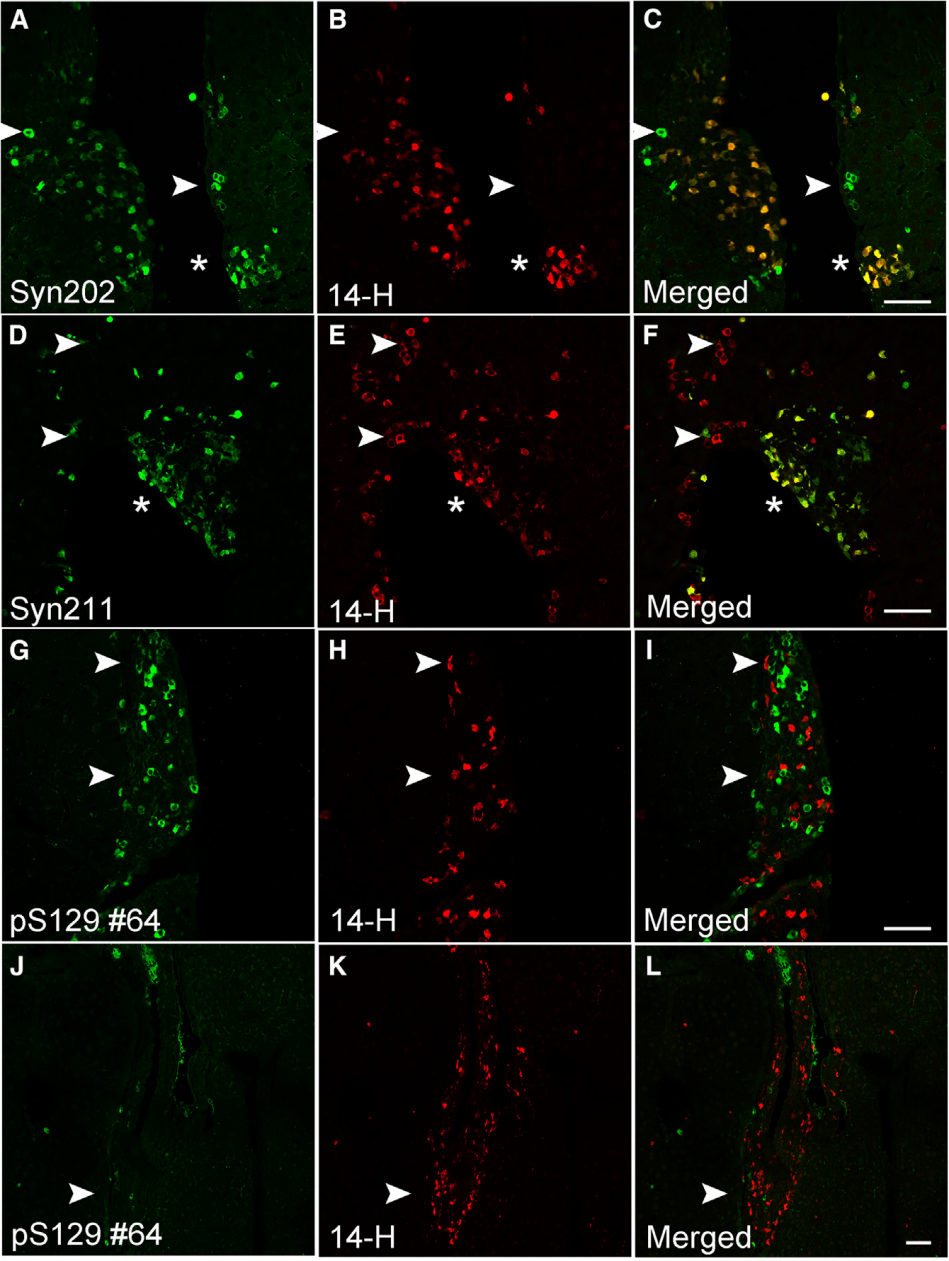
Human α-syn inclusions in the A30P liver are partially pS129-positive (A–C) Identification of human α-syn deposits immunolabeled with the Syn202 and 14-H antibodies show extensive co-localization extensively in some regions (∗) whereas in other areas this co-localization is clearly absent (arrowheads). (D–F) Co-localization between Syn211 (green) and 14-H (red) antibodies (∗) and absence (arrowhead) is observed within the same region of the liver. (G–I) The pS129 (#64) antibody clone detects the presence of human α-syn inclusions in the A30P liver which fails to co-localize with the 14-H antibody (arrowhead). (J–L) No pS129 α-syn immunoreactivity (#64) was identified in the liver within α-syn inclusions labeled with the 14-H antibody (arrowhead). Bars = 50 μm.

To investigate whether livers from the transgenic A30P model of PD harbor key pathological modifications associated with the PD brain, we performed double-labeled immunofluorescence on liver tissue sections from 18-month mice using antibodies targeting different epitopes spanning the human α-syn molecule (Figure 1; Table 1). First, we tested several widely used pan α-syn antibodies, including Syn202 which targets the C-terminal end of the molecule at amino acids 130–140 (Figure 1 and Table 1). Consistent with the sporadic distribution of human α-syn within the liver in regions near the portal and central veins,^10^ we observed Syn202-positive inclusions (in terms of abundance, +++) which co-localized with 14-H (++), an antibody that targets amino acids 117–125 (Figures 2A–2C, stars). In some instances, we also noted the presence of Syn202-positive α-syn inclusions devoid of 14-H immunoreactivity (Figures 2A–2C, arrowheads), pointing to the presence of C-terminal modification(s) within or near the 14-H epitope which abolishes antibody reactivity (Figure 1). Consistent with our previous results,^10^ the α-syn pathology observed in the liver localized primarily to hepatocellular structures as shown by the co-localization between 14-H and albumin antibodies (Figures S1A–S1I), a specific marker for hepatocytes. Importantly, we observed no human α-syn immunoreactivity in livers from wild type mice, pointing to the specificity of these antibodies to human α-syn (Figure S1).

**Table 1:** Qualitative observation of α-syn modification and their occurrence in the A30P mouse liver

α-syn Antibody	Clone	Epitope	Occurrence^a^
14-H	14H2L1	117–125	++
Syn211	Syn211	121–125	+++
Mouse α-syn	D37A6	Glu105	+
Syn202	Syn202	130–140	+++
pS129	psyn#64	pS129 (124–134)	++
pS129	Ab59264	NA	±
pS129	EP1536Y	NA	–
pY39	BS-9587R	pY39	+
nY39	nSyn14	nY39	+
PA1	PA1-18256	11–27	++
42/α-syn	42/α-syn	15–123	++
LS-B116408	LS-B116408	1–60	+
NACP112	NACP112	62–112	++
BS009	BS-0009R	51–90	±
PA5	PA5-13397	75–104	++
α-*syn*-pS87	PAB0826-p	pS87	+
LBS1185	LSB1185/60761	91–102	+
4B12	4B12	103–108	++
pY125	BS-5627R	pY125	–
pY133	BS-5625R	pY133	+
X-122	A15127A	Cleavage at 122	+
MJFR-14-6-2	AB209538	127-140/Conformation	++

a Qualitative density of α-syn pathology observed in the liver.

To further investigate the lack of 14-H immunoreactive α-syn inclusions, we stained liver sections with Syn211, another widely validated C-terminal antibody that targets amino acids 121–125 of α-syn, an epitope that partly encompasses the 14-H sequence (117–125) (Figure 1 and Table 1). To our surprise, given the immediate proximity of these two epitopes, we only observed partial co-localization between Syn211 (+++) and 14-H (Figures 2D–2F, asterisk), further suggesting the presence of PTMs which prevent 14-H binding. On the other hand, we also observed 14-H α-syn inclusions devoid of Syn211 immunoreactivity (Figures 2D–2F, arrowheads), suggesting truncation events which likely eliminate Syn211 binding.^32^ To identify potential PTMs within the C-terminal region of α-syn, we next immunostained liver sections with an antibody targeting phosphorylation at serine 129 (pS129 *#64*), a modification within close proximity to the 14-H epitope and a pathological hallmark of PD (reviewed in^33^). While we observed the presence of liver α-syn inclusions positive for pS129 (+) (Figures 2G–2I), none co-localized with the 14-H antibody, suggesting that either the negative charge or conformational changes following S129 phosphorylation hinder the 14-H epitope (Figures 2G–2I, arrows). As expected, 14-H-positive α-syn inclusions devoid of pS129 immunoreactivity were also observed, highlighting the presence of non-phosphorylated (pS129) α-syn (Figures 2J–2L, arrowhead).

To rule out a potential hindering effect or epitope masking by either antibody due to the close proximity of these epitopes, we immunostained liver tissue sections first with pS129 (clone *#64*) for 24 h and then subsequently added 14-H for an additional 24 h (Figures S2A–S2C). In parallel, we reversed the addition of antibodies so that tissues were labeled first with 14-H for 24 h followed by pS129 for the same time period (clone *#64*) (Figures S2D–S2F). Consistent with our results above, the presence of α-syn inclusions positive for pS129 and 14-H were identified. However, no co-localization was observed regardless of whether these two antibodies were added sequentially or at the same time as one another (Figures S2A–S2F). These results suggest that pS129 phosphorylation likely masks the 14-H epitope and hinders its binding affinity to α-syn inclusions in the A30P mouse liver.

**Figure 3:**
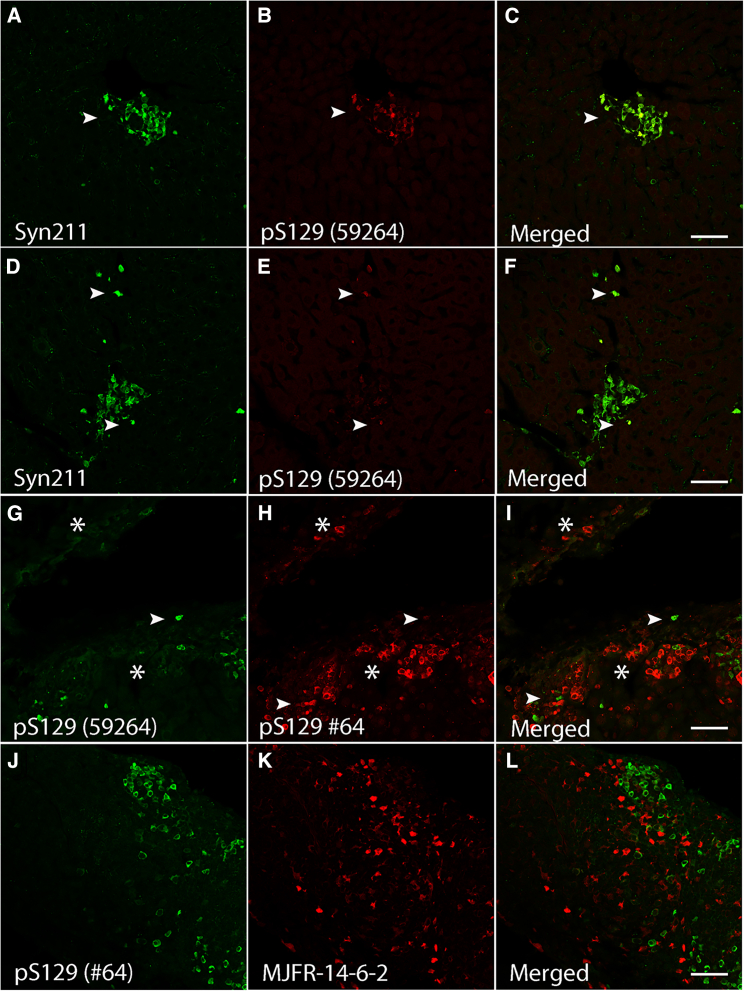
Differential binding of pS129 antibodies to human α-syn aggregates in the A30P liver (A–C) Partial co-localization (arrowhead) is observed between Syn211 and pS129 clone 59264. (D–F) Abundant Syn211-positve α-syn inclusions are rarely labeled with the pS129 (clone 59264) antibody (arrowheads). (G–I) Rare co-localization is observed (arrowhead) with the pS129 antibodies clone 59264 and pS129 #64 as both antibodies appear to label independent inclusions (∗). (J–L) pS129 (#64) α-syn inclusions in the liver do not co-localize with the conformation specific MJFR-14-6-2 antibody. Bars = 50 μm.

Given the sensitivity of pS129 antibodies to other post-translational modifications^28^ and the lack of co-localization between 14-H and pS129 (clone *#64*) antibodies, we tested other commercially available pS129 antibodies-clones Ab59264 and EP1536Y (Table 1). In contrast to pS129 (clone *#64*), limited liver α-syn inclusions positive for pS129 (±) were observed using clone Ab59264. Nonetheless, these inclusions co-localized with Syn211, confirming that a subset of α-syn aggregates within the liver are indeed phosphorylated at S129 (Figures 3A–3C, arrowheads). In addition, and consistent with the immunoreactivity of 14-H to non-phosphorylated proteins (S129), Syn211-positive α-syn inclusions devoid of pS129 immunoreactivity were also observed (Ab59264), corroborating the presence of non-pS129 modified α-syn proteins (Figures 3D–3F, arrowheads). In accordance with the observation that EP1536Y has been reported to immunoreact with pS129 α-syn in liver aggregates in patients diagnosed with non-alcoholic steatohepatitis (NASH),^34^ limited pS129 immunoreactive α-syn inclusions were observed in the A30P mouse liver (data not shown). Importantly, none of the pS129 antibodies used in this study immunolabeled α-syn pathology in wild type mice.

Given the marked differences in immunoreactivity between the two pS129 antibodies (clone #64 and Ab59264), we investigated whether these probes target the same liver-α-syn inclusions. Thus, we took advantage of their different host species (mouse and rabbit, respectively) and immunolabeled liver sections with both pS129 antibodies at the same time. While we detected α-syn inclusions positive for pS129 (clone *#64*) (Figures 3G–3I, asterisks) limited α-syn inclusions were labeled with pS129 (Ab59264) (Figures 3G–3I, arrowheads). However, both pS129 antibodies failed to immunoreact with the same α-syn inclusions, suggesting a potential hindering effect due to both antibodies targeting the same epitope (Figures 3G–3L). To validate this observation, we immunolabeled liver sections first with clone *#64* for 24 h, followed by clone Ab59264 for the same time period. In parallel, we reversed the antibody addition and immunolabeled adjacent tissue sections first with Ab59264 followed by clone #64. Consistent with our results above, no marked differences in immunostaining or co-localization between the two pS129 clones were observed, regardless of whether the antibodies were added sequentially or at the same time as one another (Figures S3A–S3F). The lack of increased immunoreactivity or co-localization with these antibodies is consistent with the idea that both probes may be differentially affected by modifications surrounding their specific epitopes,^28^ thus targeting different α-syn inclusions in the A30P liver.

Subsequent to the observations above, we investigated whether pS129-positive α-syn liver inclusions (clone #64) are immunoreactive for MJFR-14-6-2, an antibody targeting aggregated α-syn. While our results revealed the presence of MJFR-14-6-2-positive α-syn inclusions in portal tracts and central veins (++), no co-localization between MJFR-14-6-2 and pS129 (clone #64) was observed (Figures 3J–3L). These results suggest that the pS129 modification may precede the aggregation state recognized by MJFR-14-6-2. This is in line with our previous results which indicate that liver α-syn inclusions are early aggregates due to their specific interaction with luminescent conjugated oligothiophenes (LCOs).^10^^,^^35^^,^^36^^,^^37^^,^^38^ Alternatively, pS129 may prevent MJFR-14-6-2 from binding given the strong negative charge and/or the location of the linear epitope required for MJFR-14-6-2 binding, which encompasses amino acids 127–140 and falls within the pS129 epitope.^39^ Thus, these results suggest that differences in pS129 epitopes or sensitivity to surrounding modifications may influence their binding affinity limiting their reactivity *in vivo* as reported previously.^28^

**Figure 4:**
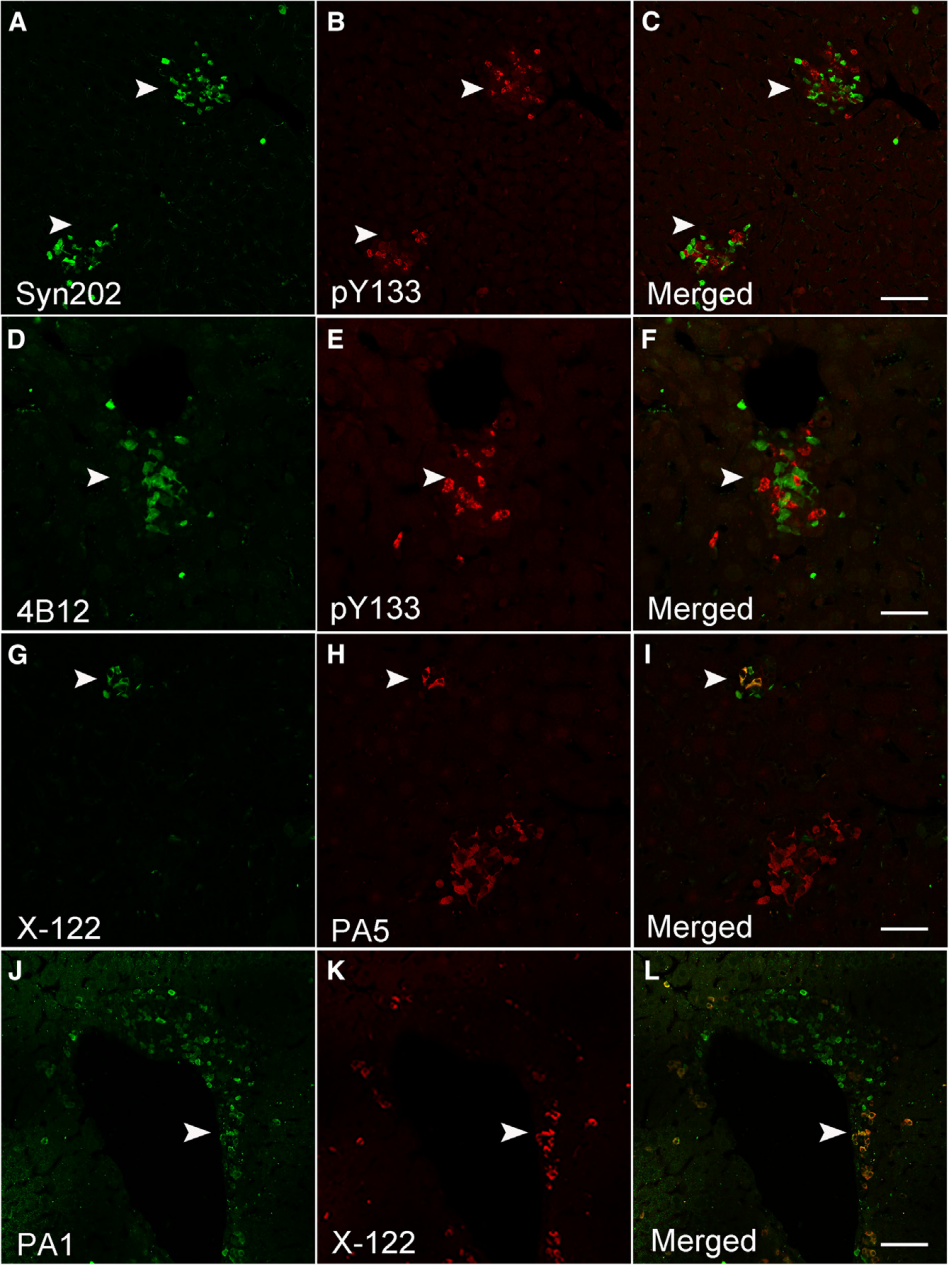
Human α-syn inclusions in the A30P liver are phosphorylated at tyrosine 133 (Y133) or truncated at position X-122 (A–C) Identification of Syn202-positive human α-syn inclusions did not co-localize with a phosphorylation specific antibody at position 133 (pY133, arrowheads) or (D–F) with the 4B12 antibody (arrowheads). (G–I) C-terminal truncation of α-syn at position X-122 is detected within the A30P liver and partially co-localizes with PA5 and (J-L) PA1 pan antibodies (arrowheads). Bars = 50 μm.

We next investigated whether other phosphorylation events within the C-terminal end of α-syn, reported to be present in the PD brain,^40^^,^^41^^,^^42^^,^^43^ are also present in the A30P mouse liver. We immunostained liver sections with commercially available phospho-specific antibodies targeting tyrosine 125 (pY125) or tyrosine 133 (pY133), located at the most C-terminal region of α-syn (Figure 1 and Table 1). In contrast to pS129, we observed no pY125 immunoreactivity in any of the livers tested, whereas sporadic α-syn inclusions immunoreactive for pY133 (±) were observed (Figures 4A–4F). However, pY133-positive inclusions did not co-localize with any of the C-terminal antibody epitopes tested including Syn211, Syn202 (Figures 4A–4C) or 4B12, an antibody targeting amino acids 103–108 of α-syn (Figure 1 and Table 1), (Figures 4D–4F, arrowhead). Our results suggest that phosphorylation at tyrosine Y133 (pY133) may also hinder the presence of C-terminal epitopes as is the case for 14-H following pS129 phosphorylation. Again, none of these immunological probes labeled α-syn in liver tissue sections from wild type mice.

We next investigated whether C-terminal truncation events are also a prominent feature of α-syn inclusions in the liver. Although multiple cleavage sites have been reported within the C-terminal end of α-syn,^30^^,^^32^^,^^44^^,^^45^^,^^46^^,^^47^ cleavage at position at X-122 is the only commercially available antibody for α-syn truncation we could identify. Given the absence of C-terminal epitopes following α-syn X-122 truncation, we immunostained tissue liver sections with X-122 and PA5, an antibody specific to the NAC region of α-syn (62–112, Figure 1 and Table 1). Although we observed limited α-syn inclusions cleaved at 122 (+), they were positive for PA5 (++) (Figures 4G–4I, arrowhead). We next stained sections with X-122 and PA1, an N-terminal α-syn antibody specific for amino acids 11–27 (Figure 1 and Table 1). Consistent with our results above, we observed a partial co-localization between PA1 (++) and X-122 (+) (Figures 4J–4L, arrowhead). However, compared to C-terminal antibodies, limited α-syn reactivity was observed with PA1, suggesting that the N-terminal region of α-syn may also be susceptible to PTMs, including nitration and phosphorylation (reviewed in^28^^,^^30^). Taken together, our observations suggest that, unlike pS129, phosphorylation at Y133 and truncation at position X-122 may be sporadic events in the aged A30P liver, whereas phosphorylation at Y125 was not identified.

### Human α-syn aggregates in the (Thy-1)-h[A30P] liver are N-terminally modified

**Figure 5:**
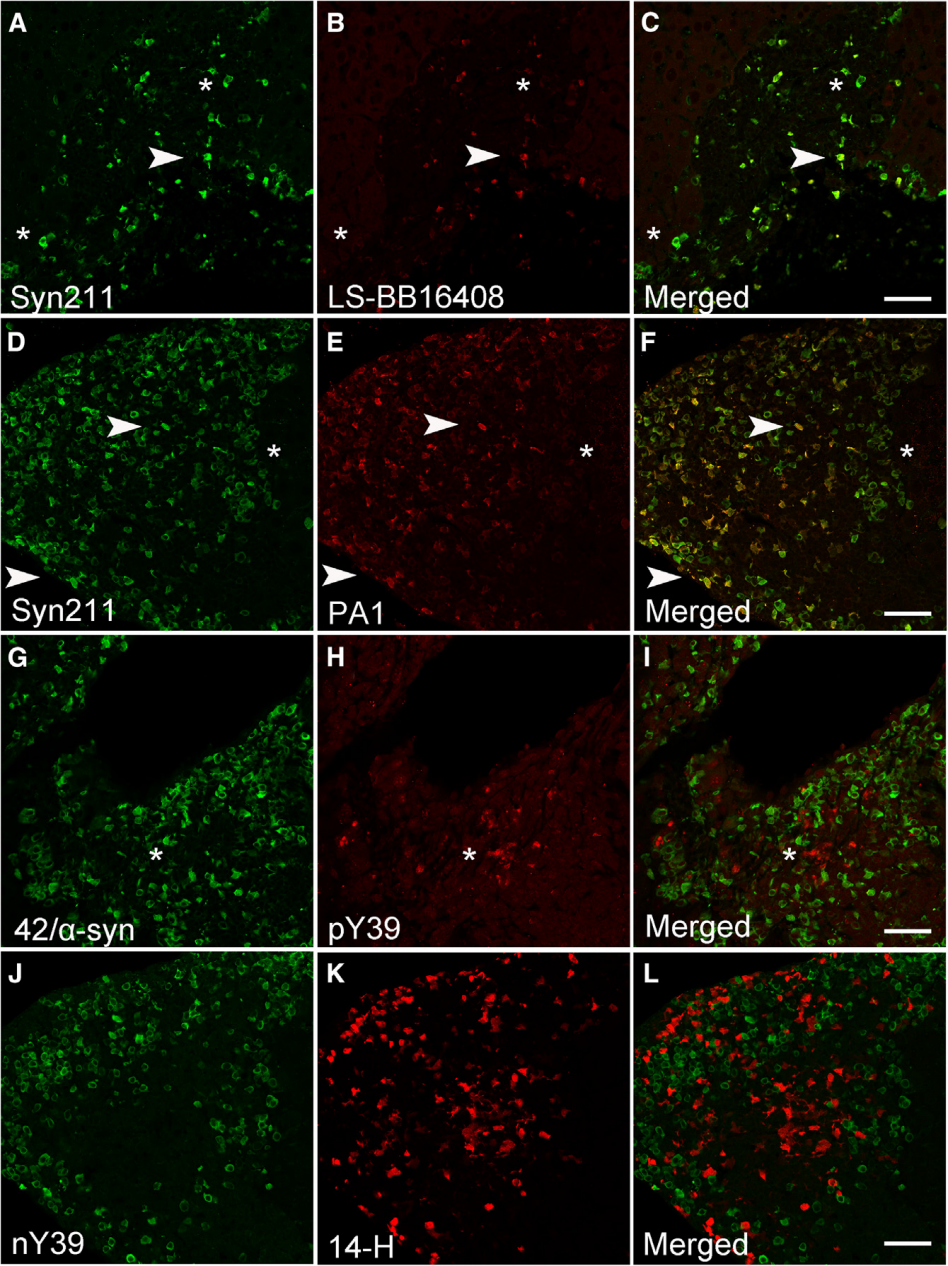
Nitration and phosphorylation at position Y39 occur within human α-syn inclusions in the A30P liver (A–C) Abundant Syn211 immunoreactivity human α-syn inclusions which rarely (∗) co-localized with the N-terminal LS-BB16408 (arrowhead). (D–F) Clear Syn211 immunoreactive human α-syn inclusions are present within the A30P liver and are partly labeled (arrowhead) with the N-terminal antibody PA1 but mostly remain unlabeled (∗). (G–I) Phosphorylation of human α-syn inclusions at pY39 fails to co-localize with the 42/α-syn (∗) (J-L) Site-specific nitration at position Y39 (nY39) within α-syn fails to co-localize with the C-terminal antibody 14-H. Bars = 50 μm.

To investigate the presence of N-terminal modifications in liver α-syn aggregates, we immunostained liver sections with an antibody raised against epitope 1–60, *i.e*., the entire N-terminal region of α-syn (LS-B816408) (Figure 1 and Table 1). While our observations revealed sparse α-syn immunoreactivity, these inclusions partly colocalized with Syn211 (Figures 5A–5C, arrowhead). Moreover, Syn211-positive inclusions devoid of LS-B816408 were also noted (Figures 5A–5C, asterisk). We then narrowed our N-terminal epitope using PA1 (11–27) and in contrast to LS-B816408, the presence of α-syn inclusions immunoreactive for PA1 and Syn211 were clearly noted (Figures 5D–5F, arrowheads). Consistent with the results above, Syn211 inclusions devoid of PA1 immunoreactivity were also identified, further suggesting the presence of N-terminal modification(s) at or near the PA1 epitope hinder antibody binding (Figures 5D–5F, asterisk). Thus, we then used the phosphorylation specific antibody targeting tyrosine 39 (pY39), a modification identified in normal as well as PD brains and reported to have a role in α-syn aggregation.^31^ Although our results revealed the presence of pY39 α-syn inclusions (+), these did not co-localize with Syn211 (data not shown) or 42/α-syn (++), another α-syn antibody reportedly raised against amino acids 15–123 (Figures 5G–5I, asterisk). We next investigated tyrosine nitration within the same residue (nY39). Our results revealed partial co-localization between nY39 and PA1, confirming the presence of nitrated α-syn within the liver (data not shown). Interestingly, the nY39-positive inclusions observed were devoid of 14-H immunoreactivity, suggesting the presence of pS129 phosphorylation or C-terminal truncation events which eliminate 14-H binding (Figures 5J–5L). Taken together, our results demonstrate for the first time that a subset of α-syn inclusions within the liver are modified at both N- and C-terminal regions of the α-syn molecule. Importantly, none of the N-terminal antibodies used here showed α-syn reactivity in wild type livers.

### The non-amyloidogenic component region of α-syn in the (Thy-1)-h[A30P] liver is selectively modified

**Figure 6:**
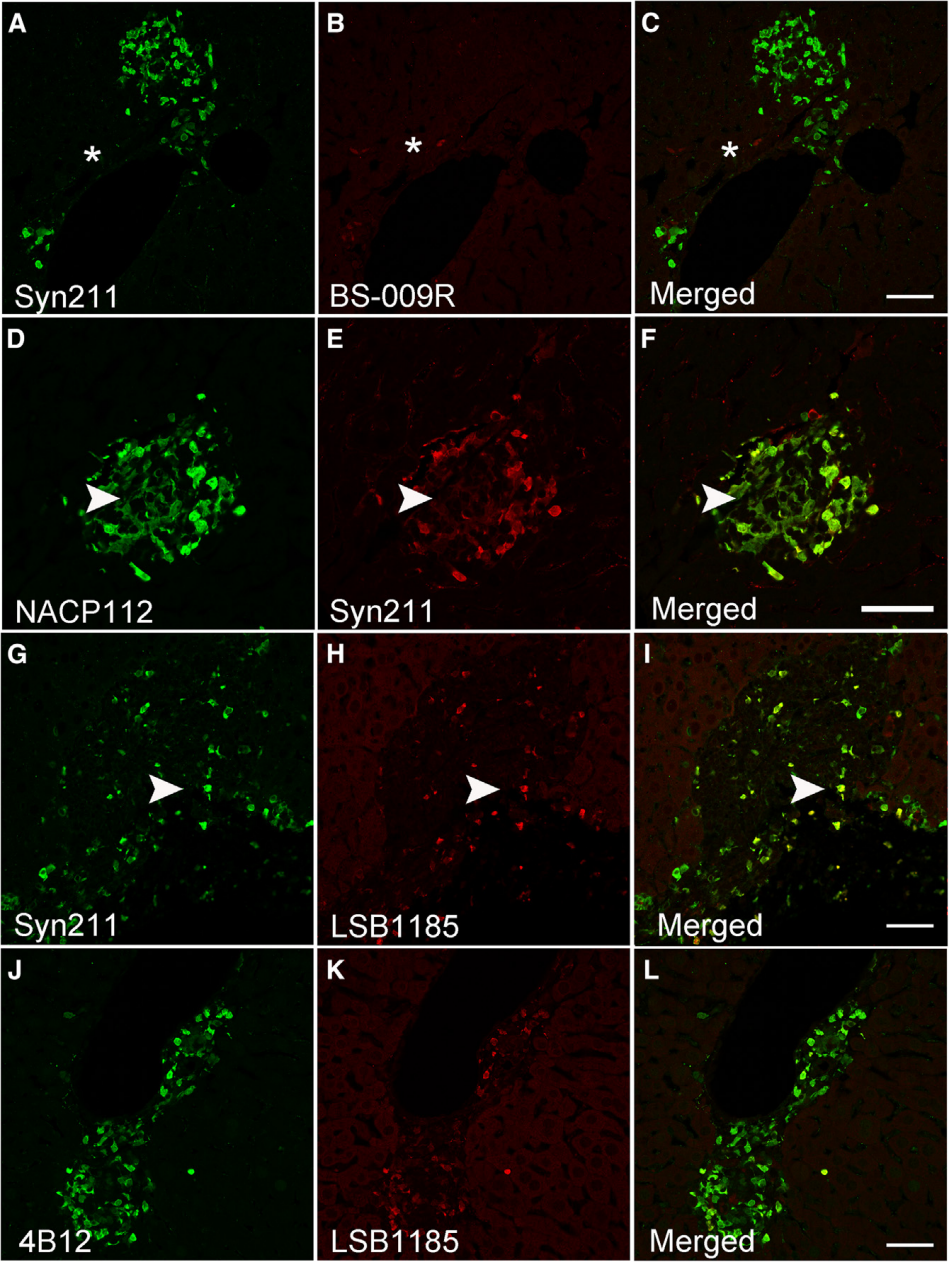
Limited N-terminal α-syn reactivity was observed in the A30P liver (A–C) Rare N-terminal human α-syn immunoreactivity was detected in the liver using the pan-α-syn antibody BS-009R (∗) compared to Syn211. (D–F) Limited co-localization between Syn211 and the NACP112 antibodies (arrowhead). (G–I) Partial co-localization observed with Syn211 and LSB1185 antibodies (arrowhead). (J–L) Rare identification of LSB1185-positive human α-syn inclusions that co-localized with the 4B12 antibody. Bars = 50 μm.

**Figure 7:**
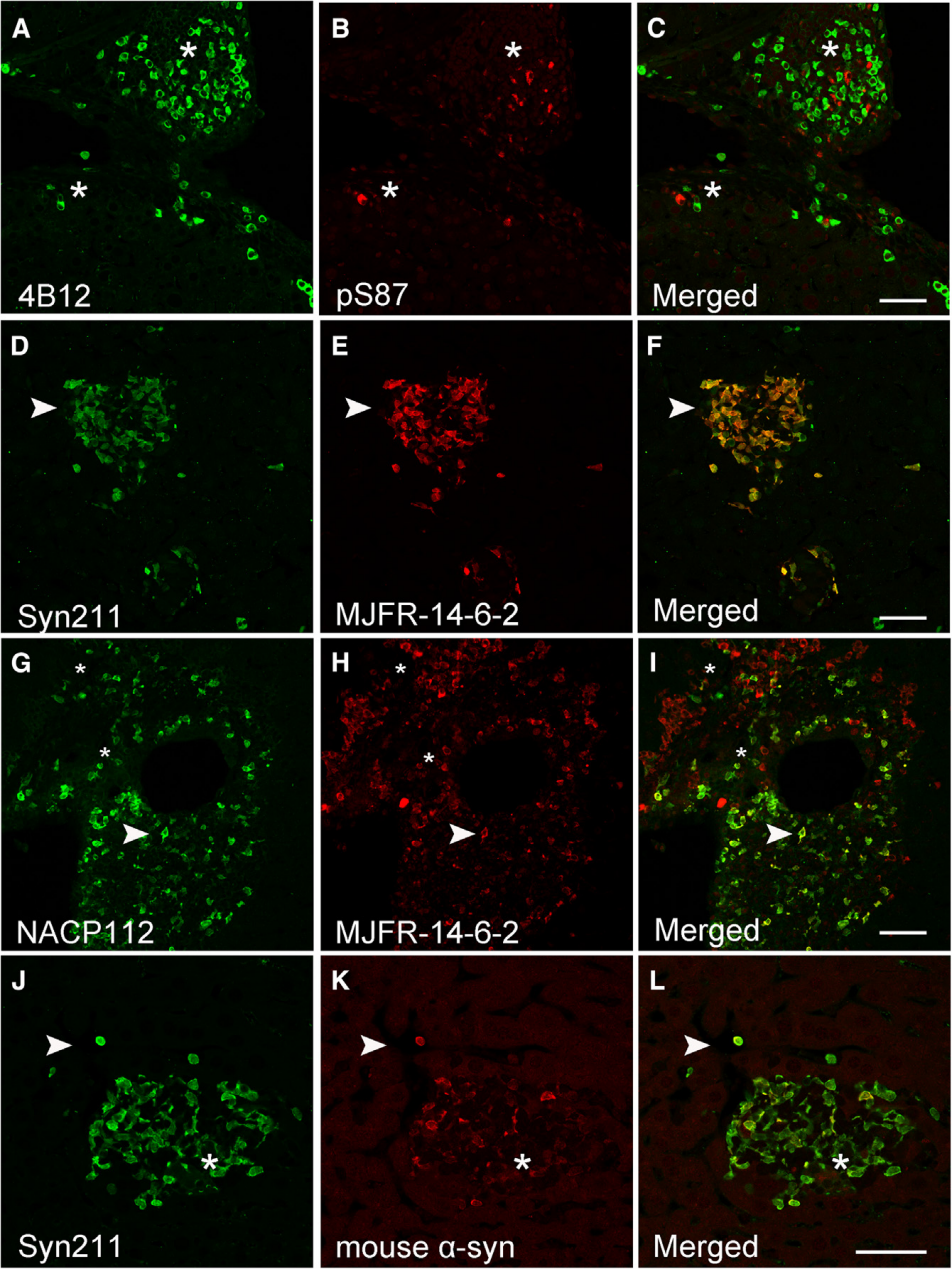
Human α-syn inclusions in the A30P liver seed and co-localize with endogenous mouse α-syn proteins (A–C) Liver α-syn aggregates are phosphorylated at serine 87 (pS87) and fail to co-localize with the pan-α-syn antibody 4B12 (asterisk). (D–F) Identification of Syn211 inclusions which co-localize with the misfolded-specific antibody MJFR-14-6-2 and with the G-I) NACP112 antibody (arrowheads). (J–L) Identification of Syn211-positive human α-syn inclusions which partly co-localize (arrowhead) with endogenous mouse-specific α-syn antibody, while some appear to be completely negative (∗). Bar = 50 μm.

We next assessed the accessibility of α-syn epitopes within the non-amyloidogenic component (NAC) of α-syn using multiple antibodies including BS-009R, an antibody raised against amino acids 51–90 of α-syn (Figure 1; Table 1). Although our results revealed abundant α-syn pathology with Syn211, limited immunoreactive α-syn inclusions were detected using BS-009R (±), (Figures 6A–6C, asterisk). We next tested NACP112, an antibody raised against amino acids 62 to 112 (Figure 1 and Table 1). Unlike BS-009R, NACP112 revealed marked α-syn inclusions (++) that were also Syn211 positive (Figures 6D–6F, asterisk). However, NACP112-positive α-syn inclusions devoid of Syn211 were also noted, suggesting the presence of C-terminal truncation events (X-122) which will eliminate Syn211 immunoreactivity (Figures 6D–6F, arrowhead). We next immunostained tissue sections with LSB1185, an antibody whose epitope resides between amino acids 91–102 (Figure 1 and Table 1). Although limited α-syn inclusions immunoreactive for LSB1185 were detected (+), they were positive for Syn211 (Figures 6G–6I, arrowhead) and 4B12 (Figures 6J and 6K). Finally, we assessed phosphorylation at serine 87 (pS87), a modification reported to occur in the brains of healthy controls and PD cases.^48^ Although our results revealed sparse pS87-positve α-syn inclusions within the liver, these were negative for Syn211, Syn202 or 4B12 (Figures 7A–7C, asterisks). Notably, no pS87 reactivity was observed in wild type livers. Finally, we assessed whether the α-syn inclusions labeled with pan α-syn antibodies colocalize with MJFR 14-6-2, an aggregation dependent antibody. As expected, MJFR 14-6-2 inclusions co-localized with Syn211 (Figures 7D–7F, arrowhead), 4B12 (data not shown), and NACP112 (Figures 7G–7I, arrowhead). Noteworthy is the finding of NACP112 positive α-syn inclusions lacking MJFR-14-6-2 immunoreactivity (Figures 7G–7I, asterisk) which point to the presence of pS129 or to a potential cleavage event that would eliminate the linear epitope required for MJFR-14-6-2 binding.^39^

In view of our recent findings, which indicate that neither the endogenous mouse nor human *SNCA* genes are expressed in the liver of WT or transgenic A30P mice,^10^ we investigated whether endogenous mouse α-syn is recruited to the liver and co-aggregates with human α-syn inclusions. Thus, we immunostained liver tissue sections with a specific antibody for endogenous mouse α-syn targeting amino acids surrounding Glu105 (Figure 1; Table 1). Our results revealed the presence of mouse α-syn inclusions which co-localized in part, with the human specific antibody Syn211 (Figures 7J–L, arrowheads). Taken together and given the lack of endogenous expression of mouse α-syn in the liver of A30P mice, our results suggest that mouse α-syn co-aggregates with human α-syn inclusions in the brain before they are transported to the liver for clearance.^10^

### Human hepatocytes differentially cleave α-synuclein fibrillar assemblies and oligomeric assemblies *ex vivo*

**Figure 8:**
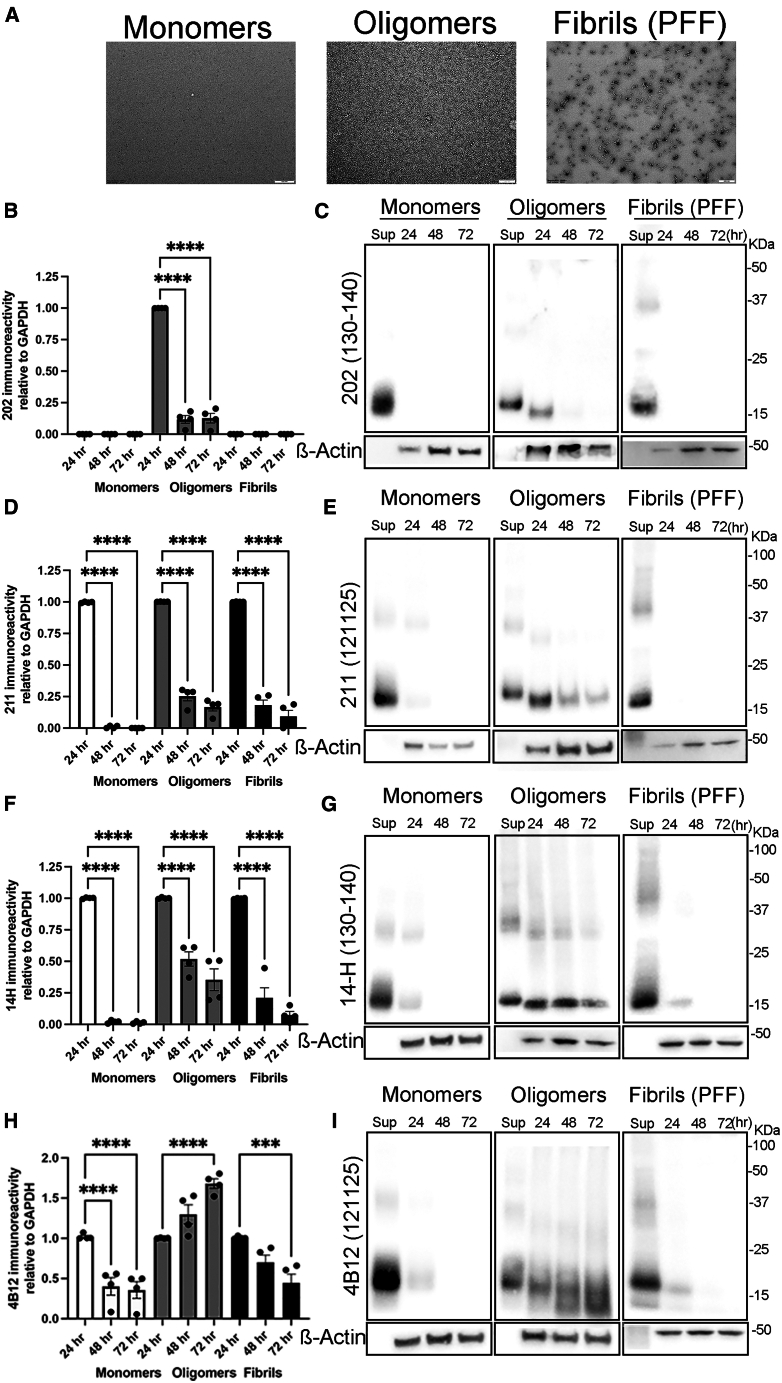
Differential processing of human α-syn assemblies by human Huh-7 hepatocytes *in vitro* (A) Transmission electron microscopy (TEM) of α-syn monomers, oligomers, and fibrillar assemblies showing the classical donut-like and filamentous ultrastructures, respectively. Bar = 200 nm (B–C) Quantification of human α-syn immuno-reactivity in HuH-7-Cx32 cells at different time periods (24, 48 and 72 h) or in the supernatant (Sup, 72 h) using Western blot analysis with the Syn202, (D-E) Syn211, (F-G) 14-H, and H-I) 4B12 antibodies and analyzed using one-way ANOVA with Tukey’s multiple comparison test. Data are represented as SEM and a number of replicates (*n)=4,* ∗∗∗*p* = 0.001, ∗∗∗∗*p* = 0.0001, respectively.

We next investigated the capacity of liver hepatocytes to degrade α-syn assemblies *ex vivo*. Thus, we generated α-syn monomers, oligomers (oα-syn) and fibrillar assemblies (PFFs) and then characterized them as we previously reported.^49^ Similar to our previous reports, we identified round-like and filament ultrastructure typical of oα-syn and PFF assemblies, respectively (Figure 8A).^50^ Following α-syn assembly validation, we treated the human hepatocyte cell line Huh-7 overexpressing Cx32 (Huh-7-Cx32) which promotes α-syn uptake,^10^ with all three different protein assemblies generated *in vitro*. Subsequently, we assessed protein degradation by monitoring the disappearance of the C-terminal epitopes by Western blot analysis at different time points (24, 48, and 72 h). Our results revealed that monomeric and sonicated PFF assemblies are effectively cleaved within 24 h at the most C-terminal region of the molecule, as shown by the complete disappearance of the Syn202 epitope (130–140). In contrast, the same epitope remains relatively intact in oligomeric assemblies, although at a noticeably lower molecular weight compared to the control (Sup), suggesting a potential truncation event at another region (Figures 8B and 8C). Similarly, when we assessed for the presence of the Syn211 epitope (121–125) we observed its complete disappearance from monomers within 48 h. However, its presence was only slightly decreased in both fibrillar and oligomeric assemblies (Figures 8D and 8E). Correspondingly, the 14-H epitope disappeared within 48 h upon incubation with monomers, whereas the same epitope persisted at 72 h in oligomer and PFF assemblies (Figures 8F and 8G). Finally, we assessed the 4B12 epitope (103–108) and consistent with the results above, it decreased in both monomeric and PFF α-syn assemblies with the lowest reactivity at 72 h (Figures 8H and 8I). However, the 4B12 epitope increased over time in Huh-7-Cx32 hepatocytes exposed to oligomeric assemblies, with the most pronounced effect at 72 h. Notably, the appearance of two molecular weight bands lower than the intact original proteins (Sup) was observed (Figures 8H and 8I). It is worth mentioning that these protein bands appear within the approximate molecular weight as those recently reported by Quin and colleagues following truncation at X-103 and X-114.^44^ Taken together, our results indicate that human hepatocytes can differentially process the C-terminal end of α-syn assemblies, a mechanism that likely depends on the conformational state of the molecule.

### Activation of autophagy increases oligomeric α-synuclein degradation in human hepatocytes

Given that oligomeric α-syn assemblies are more resistant to degradation due to the intact C-terminal epitopes compared to monomers or PFFs, we investigated whether enhancing autophagy using pharmacological inhibitors of mTOR^51^ could increase their degradation. Thus, we incubated oligomeric α-syn with Huh-7-Cx32 cells and then assessed their degradation efficiency in the presence of increasing concentrations of rapamycin, a widely used pharmacological enhancer of autophagy.^52^ Our results demonstrate that rapamycin enhances oligomeric α-syn degradation in a concentration dependent manner, as shown by the gradual disappearance of the 14-H epitope within 24 h, which at normal conditions persists longer than 72 h (Figures S4A–S4B). These results suggest that human hepatocytes possess the capacity to degrade α-syn assemblies, a process that can be enhanced by promoting autophagy.

### Human hepatocytes selectively induce pS129 modification *in vitro*

**Figure 9:**
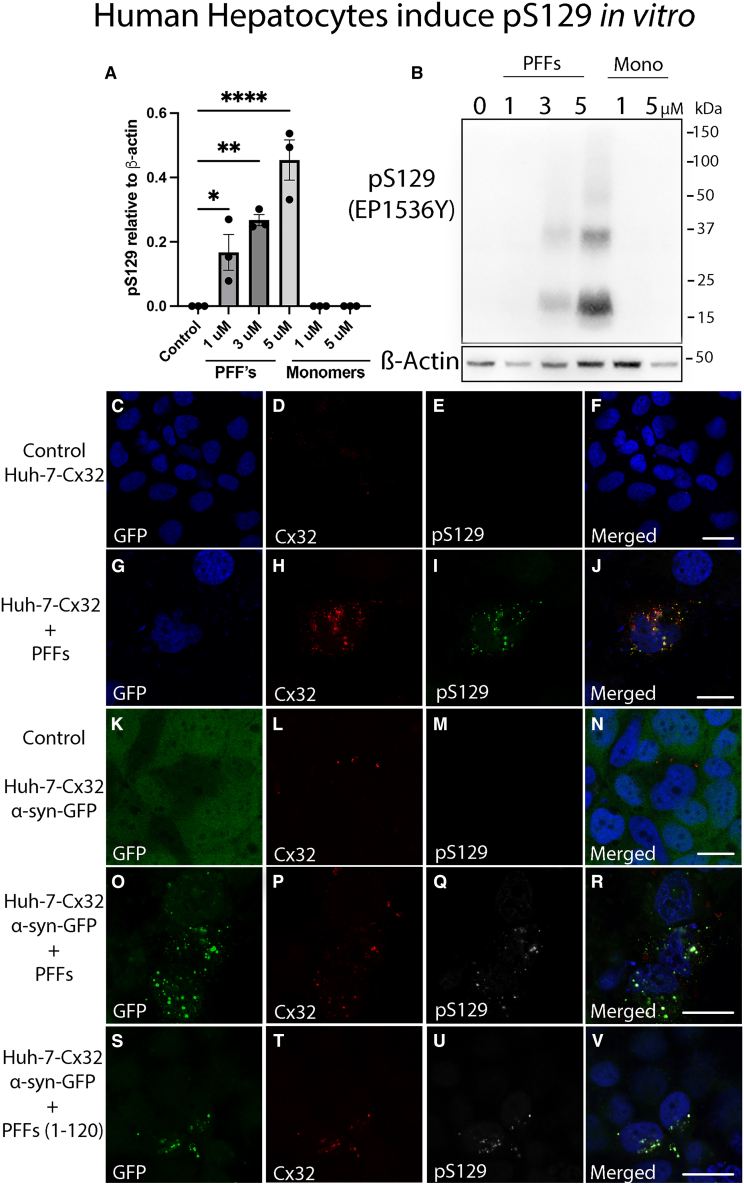
Human hepatocytes (Huh-7) induce pS129 phosphorylation *in vitro* (A–B) Western blot analysis of Huh-7 cells treated with different concentrations of PFFs or α-syn monomers (1–5 μM) for 72 h then immunoblotted for pS129 (EP1536Y) and β-actin as loading control. (C–F) Untreated Huh-Cx32 cells show no pS129 immunolabeling whereas (G-J) treated cells with full length PFF show puncta immunoreactive for pS129 and partly co-localize with Cx32-mCherry. (K-N) Untreated control Huh-Cx32-α-syn expressing cells show no pS129 immunoreactivity whereas (O-R) cells treated full length PFF show puncta immunoreactive for pS129 and partly co-localize with Cx32-mCherry. (S–V) Huh-Cx32-α-syn expressing cells treated with truncated PFF (1–120) show puncta immunoreactive for pS129 and show partial co-localization with Cx32-mCherry. Data was analyzed using one-way ANOVA with Tukey’s multiple comparison test. Data are represented as SEM and number of replicates (*n)=3,* ∗*p* = 0.05 and ∗∗*p* = 0.01, and ∗∗∗∗*p* = 0.0001, respectively). Bars = 20 μm.

To investigate whether specific post-translational modifications observed *in vivo* can be induced *ex vivo*, we treated human Huh-7-Cx32 hepatocytes with increasing concentrations of PFF assemblies for 72 h to overcome the initial degradation capacity and prevent C-terminal degradation, a timeline at which phosphorylation at serine 129 has been reported to occur *in vitro.*^53^ Our results demonstrate that, similar to neurons, human hepatocytes successfully phosphorylate exogenous α-*syn*-PFFs in a concentration dependent manner (Figures 9A and 9B). As expected, pS129 phosphorylation does not occur in the presence of α-syn monomers due to their immediate degradation by Huh-7 cells (Figure 8). We then performed confocal image analysis to validate the presence of α-*syn*-pS129 modifications. As expected, no pS129 labeling was detected in control untreated Huh-7-Cx32 cells, whereas α-*syn*-PFF treatment revealed the classical puncta structures that were, in some instances, pS129 positive and co-localized with the gap junction protein Cx32 (mCherry) (Figures 9C–9J, arrows), a transmembrane protein we previously identified to be involved in α-syn uptake and transmission.^10^^,^^49^ Importantly, under the current conditions, no pS129 reactive inclusions were observed using α-syn oligomers for up to five days of treatment (data not shown), pointing to clear structural differences between α-syn oligomers and PFFs, suggesting that longer time periods may be required for pS129 induction in the presence of α-syn oligomers.

Given the lack of α-syn expression in human hepatocytes,^10^ we next expressed WT human α-syn tagged to GFP (α-*syn*-GFP) in Huh-7-Cx32 cells and then assessed whether endogenous α-*syn*-GFP could be seeded and phosphorylated *in vitro* following PFF treatment. Thus, we treated huh-7-Cx32-α-*syn*-GFP cells with either full length or truncated PFFs which lack the C-terminal end of the molecule (1–120), fibrillar assemblies which prevent self-pS129 phosphorylation but remain seeding competent.^54^ As expected, our results demonstrate the absence of α-syn inclusions or pS129 reactivity in control untreated cells, whereas the treatment of huh-7-Cx32-α-*syn*-GFP using PFFs successfully induced the formation of α-*syn*-GFP inclusions that, in some instances were both pS129 and Cx32 positive (Figures 9K–9R). Similarly, the treatment of truncated PFFs (1–120) successfully induced the formation of α-*syn*-GFP inclusions that were morphologically indistinguishable from full-length PFFs including pS129 reactivity and Cx32 co-localization, although the α-*syn*-GFP aggregates seeded by truncated PFFs were less abundant than full-length PFFs (Figures 9S–9V). Collectively, our results suggest that human hepatocytes possess the capacity to induce specific α-syn PTMs, indicating that not all proteins arriving at the liver are in a modified state but may be modified upon arrival. Thus, our hepatocyte cellular model could facilitate the identification of other modifications observed in the mouse liver, pinpointing a novel role for the liver in the induction of PTMs of α-syn assemblies which may be necessary for its clearance and detoxification.

## Discussion

In an earlier report, we described for the first time the accumulation of pathological α-syn assemblies in the liver of multiple mouse models of PD and MSA, expressing human α-syn under the control of CNS-specific promoters.^10^ Similar to the brain, protein deposition within the liver leads to a progressive inflammatory response that was particularly evident in the A30P model at three months of age.^10^ Thus, we concluded that the progressive accumulation of human α-syn assemblies in the liver was responsible for the inflammatory response, as no detectable signs of inflammation were observed in wild type littermate controls regardless of age.^10^ In the current study, we investigated whether human α-syn liver deposits in the aged (18-month) A30P model recapitulate the hallmark PTMs associated with the PD brain. Our results revealed the presence of α-syn inclusions susceptible to different PTMs, including tyrosine nitration (nY39), site-specific phosphorylation (pY39, pS87, pS129 and pY133), C-terminal truncation (X-122) and pathological conformation events (MJFR 14-6-2), all pathological features of PD brain pathology.^28^^,^^29^^,^^30^^,^^31^ As expected, no detectable human α-syn or associated PTMs could be identified in wild type liver tissues, highlighting the specificity of the antibodies used and the specific accumulation of human α-syn pathology in the A30P model of PD. It is worth noting that we only performed co-localization experiments using antibodies raised in different hosts to avoid potential cross-reactivity, although this limited the combination of probes used in this study.

Nonetheless, we could demonstrate that a subset of modified α-syn inclusions show co-localization with a limited number of pan-synuclein antibodies including Syn202, Syn211, and 14-H within the C-terminal end, 4B12, PA5, and NACP112 within the NAC region and PA1 at the most N-terminal region of the molecule. Although we identified human α-syn inclusions in the liver to be positive for MJFR-14-6-2, these did not co-localize with pS129, the most characteristic pathological post-translational modification in the PD brain.^33^ The lack of co-localization between MJFR-14-6-2 and pS129 is notable, given that phosphorylation at this residue has been suggested to occur during the initial stages of α-syn aggregation as shown here and in previous studies.^33^^,^^53^ These results suggest that a subset of liver α-syn inclusions may be at the initial stages of aggregation upon arrival to the liver. These results are in line with our previous reports which suggest that the liver in the A30P model of PD contains immature human α-syn aggregates as they fail to bind Thioflavin S but interact with luminescent-conjugated oligothiophenes (LCOs), molecular probes that selectively recognize immature inclusions.^10^ Alternatively, given that MJFR-14-6-2 targets the C-terminal end of α-syn (127–140) in addition to its conformational state,^39^ we cannot rule out that pS129 phosphorylation also prevents MJFR-14-6-2 or other antibodies within the same region from binding, as is the case for 14-H and Syn202/4B12 antibodies following S129 and Y133 phosphorylation, respectively.

While we identified several phosphorylation sites to be present in liver α-syn aggregates from the A30P model of PD (Y39 and S87, S129, Y133), phosphorylation at Y125, however, was not observed. This is interesting given that phosphorylation at Y125 as well as Y39 appears to be present in PD cases as well as in the L61 model of PD over-expressing human wild type α-synuclein,^31^ a model we previously identified to also contain liver α-syn pathology.^10^ Other studies, however, have reported that there is no Y125 phosphorylation in PD cases^55^ or that it is reduced with aging and/or disease progression,^56^ explaining in part, its absence within liver α-syn aggregates in the aged A30P model. Nonetheless, it is possible that the pY125 antibody used in this study may be affected by surrounding modifications, including phosphorylation and/or C-terminal truncation events, which will likely eliminate this epitope.^44^^,^^47^

The identification of α-syn phosphorylation at serine 87 (pS87) is noteworthy, given that phosphorylation at this residue has been identified in PD, mouse models of PD, dementia with Lewy bodies (DLB) as well as MSA,^48^ albeit with conflicting results.^57^ Nonetheless, phosphorylation at this residue has been postulated to have a protective effect on α-syn aggregation both *in vitro* and *in vivo.*^58^ On the other hand, limited studies have reported phosphorylation at Y133 in PD,^55^^,^^59^ potentially due to a truncation event at this position which will likely eliminate such epitope.^60^ Thus, additional studies are required to validate the role of pY133 or its truncation counterpart on the progression of PD. Finally, while we also identified nitration at tyrosine 39 (nY39) in liver α-syn inclusions, this modification has also been reported within the hallmark pathology of PD.^61^ *In vitro,* nY39 modification has been suggested to increase α-syn aggregation and decrease its binding to synthetic vesicles.^62^ It is worth noting that inflammation resulting from the progressive deposition of α-syn can lead to oxidative and nitrative modifications in the brain, as we previously reported,^63^^,^^64^^,^^65^ pointing to inflammation in the liver as a potential event that may also promote nitrative α-syn modifications. However, we cannot rule out that the nY39 nitration occurs in the brain before modified α-syn is transported to the liver.^10^ Given that neither human nor endogenous α-syn are expressed in the liver of these transgenic mice,^10^ we speculate that α-syn assemblies may be transported from the brain to the liver via the blood circulation. However, it remains unknown how α-syn proteins can escape proteolysis and reach the liver at least partially intact, as indicated by the reactivity of N- (PA1) and C-terminal antibodies (Syn202, 14H). Recent work from Kluge and colleagues demonstrated that α-syn is present in exosomes extracted from the blood of patients with PD and such α-syn assemblies are seeding competent.^66^ Thus, CNS-derived α-syn may be transported to the liver via the circulation system either within extracellular vesicles or as free-floating proteins, some of which may end up as liver aggregates following blood filtration.^10^

Consequent to the identification of α-syn modifications *in vivo*, we investigated whether human hepatocytes efficiently modify and degrade α-syn assemblies *in vitro*. We observed that human hepatocytes successfully phosphorylate α-*syn*-PFFs at serine 129. However, we could not detect pS129 modifications following oligomeric treatment under the same experimental conditions. This observation suggests that differences in protein structural assembly may delay or expediate different molecular processes that lead to different protein modifications. Indeed, to our surprise, monomers and fibrillar assemblies could be more efficiently degraded by human hepatocytes compared to α-syn oligomers. However, by using the pharmacological activator of autophagy, rapamycin, we successfully increased oligomeric α-syn degradation in a concentration dependent manner. Although other degradation pathways have been implicated in the degradation and clearance of α-syn assemblies,^67^^,^^68^^,^^69^^,^^70^ dysfunction in any of these mechanisms could promote the accumulation and/or spreading by multiple pathways including exosomes and nanotubes (reviewed by us and others^71^^,^^72^^,^^73^). These results suggest that the enhancement of the degradation mechanisms which are impaired in multiple neurodegenerative disorders,^74^ may provide a suitable therapeutic strategy to enhance protein aggregate clearance.

The efficient removal of the C-terminal epitopes from both monomers and fibrils is consistent with the absence of C-terminal α-syn epitopes including Syn202, Syn211, and 14H. In oligomers, however, we noted a limited decrease in the presence of these epitopes while an increased presence of the 4B12 epitope over time. This observation is in accordance with a recent report which indicates that C-terminal cleavage of α-syn occurs predominantly at position X-103 and X-114 *in vitro,*^44^ coinciding with the increase in the 4B12 epitope and the generation of two protein fragments following truncation. However, it is also possible that liver hepatocytes process α-syn assemblies differently and can degrade these more efficiently than neurons or other CNS-derived cell types. Given that humans and rodents lack α-syn expression in the liver,^10^ the self-seeding ability of exogenous proteins is limited. However, liver cells can efficiently modify α-syn aggregates even in the absence of endogenous expression, as shown in our hepatocyte model system. This suggests the possibility that α-syn aggregates can arrive at the liver either in a modified state or be modified upon arrival, pinpointing a novel role for the liver in the induction of α-syn PTMs necessary for their clearance and/or detoxification.^10^

Taken together, our results demonstrate that key pathological α-syn PTMs associated with the PD brain are also present within the liver in the A30P model of PD. As the liver of these mice does not express human α-syn, these species are most likely transported from the brain, either directly or indirectly via another peripheral source, in an already modified state or modified upon arrival. Thus, further investigation is needed to shed light on the underlying mechanisms related to liver α-syn pathology and its relationship to PD and other synucleinopathies.

### Limitations of this study

In this article, we demonstrate that human α-syn aggregates present in the liver contain key post-translational modifications associated with PD despite the lack of human or mouse α-syn expression in this organ. However, this study could benefit from validating the presence of these modifications in human PD livers to pinpoint the relevance of these modifications in the progression of PD. In addition, the validation of the circulatory system as the route of α-syn transmission from the brain to the liver could confirm a role for the liver in the progression of PD. Despite these limitations, we present evidence suggesting that the liver may play a major role in the clearance of PD-associated pathology derived directly from the brain or indirectly from different peripheral tissues.

## Resource availability

### Lead contact

Requests for further information and resources should be directed to and will be fulfilled by the lead contact, Juan F. Reyes (juan.reyes@liu.se).

### Materials availability

All unique/stable reagents generated in this study are available from the lead contact with a completed material transfer agreement.

### Data and code availability

• Data: All data reported in this article will be shared by the lead contact upon request. Any additional information required to reanalyze the data reported in this article is available from the lead contact upon request.
• Code: This article does not report the original code.

## Acknowledgments

We thank Dr. Maria Ntzouni and Dr. Vesa Loitto from the histology and imaging facilities at Linköping University for technical assistance. The authors gratefully acknowledge the financial support from the 10.13039/501100004359Swedish Research Council (2019-01016), The Swedish Brain foundation, The Östergötland Research Foundation for Parkinson’s Disease, The Swedish Dementia Foundation, The Swedish Parkinson's Foundation, The 10.13039/501100005701Åhlén Foundation, and Åke Wibergs stiftelse. Funding agencies were not involved in the design or interpretation of the study.

## Author contributions

JFR conceived and performed the study and wrote the original article. JFR and MH analyzed and discussed the results and edited the article. SEL, MI, and PK supervised the (Thy-1)-h[A30P] α-syn animal studies. All authors were involved in the discussion of the results and editing of the final version of the article.

## Declaration of interests

MI is a paid consultant to BioArctic AB.

## STAR★Methods

### Key resources table

**Table:** 

REAGENT or RESOURCE	SOURCE	IDENTIFIER
**Antibodies**		

14-H	Thermo Scientific	14H2L1, RRID: AB_2532379
Syn211	Abcam	Syn211, RRID: AB_1810987
Mouse α-syn	Cell Signaling	D37A6, RRID: AB_1904156
Syn202	Santa Cruz Biotech	Syn202, RRID: AB_628321
pS129	Wako Chemicals	psyn#64, RRID: AB_516843
pS129	Abcam	Ab59264, RRID: AB_2270761
pS129	Abcam	EP1536Y, RRID: AB_869973
pY39	BioLegend	BS-9587R, RRID: AB N/A
nY39	Millipore	nSyn14, RRID: AB_310821
PA1	Thermo Fisher	PA1-18256, RRID: AB_1077524
42/α-syn	BD Transduction Laboratories	42/α-syn, RRID: AB_ N/A
LS-B116408	LS-BIO	LS-B116408, RRID: AB_ N/A
NACP112	FabGennix	NACP112, RRID: AB_2286392
BS009	Bioss	BS-0009R, RRID:AB_N/A
PA5	Thermo Fisher	PA5-13397, RRID: AB_2192675
α-*syn*-pS87	Covalab	PAB0826-p, RRID: AB_N/A
LBS1185	LS-BIO	LSB1185/60761, RRID: AB_N/A
4B12	GeneTex	4B12, RRID: AB_380314
pY125	Bioss	BS-5627R, RRID: AB_11052563
pY133	Bioss	BS-5625R, RRID: AB_11066832
X-122	BioLegend	A15127A, RRID: AB_2650689
MJFR 14-6-2	Abcam	AB209538, RRID: AB_N/A
Albumin	Abcam	AB106582, RRID: AB_10888110
IgG goat anti-mouse Alexa Fluor 488	Life Technologies	Cat#A11029
IgG goat anti-mouse Alexa Fluor 564	Life Technologies	Cat#A11032
IgG goat anti-rabbit Alexa Fluor 488	Life Technologies	Cat#A11008
IgG goat anti-rabbit Alexa Fluor 564	Life Technologies	Cat#A11037
IgG donkey anti-sheep Alexa Fluor 488	Life Technologies	CatA11015
HRP-tagged anti-mouse IgG	Agilent Technologies	Cat#P0447
HRP-tagged anti-rabbit IgG	Agilent Technologies	Cat#P0448

**Bacterial and virus strains**		

mCherry-Cx32-7	Addgene	Addgene #55022
Alpha-synuclein-GFP	This paper	N/A

**Chemicals, peptides, and recombinant proteins**		

Full length wild type recombinant alpha-synuclein proteins	Alexotech	Cat#AS-600-100
Truncated wild type recombinant alpha-synuclein proteins (1–120)	Alexotech	Cat#AS-602-10
PBS pH 7.4 (1X)	Thermo Scientific	Cat#10010-015
Uranyl acetate	PolySciences Europe Gmbh	Cat# 21447-25
Carbon grids	TED PELLA Inc	Cat#01813-F
MEM Culture medium	Thermo Scientific	Ca#41090-028
Glutamine 200 mM (100X)	Thermo Scientific	Cat#25030-024
Penicillin/streptomycin	Thermo Scientific	Cat#15070-063
Amaxa Transfection Kit	Lonza	Cat#VCA-1001
4% Paraformaldehyde (PFA)	Santa Cruz Biotechnology	Cat#sc-281692
TrypLE Express^TM^	Thermo Scientific	Cat#12605-010
RIPA Buffer	Sigma Aldrich	Cat#20-188
Laemmli sample buffer	Sigma Aldrich	Cat#J60015
4-20% SDS-PAGE gels	Sigma Aldrich	Cat#MP42G15
Nonfat dry milk	Bio Rad	Cat#10004504
Tween-tris-buffered saline	Medicago	Cat#09-7510-100
ECL substrate	Bio Rad	Cat#170-5061
Xylene	Sigma-Aldrich	Cat#534056
Ethanol	Sigma Aldrich	Cat#1.08543
Triton X-100	Sigma Aldrich	Cat#T8787
Sudan Black	Sigma Aldrich	Cat#199664
Bovine Serum Albumin (BSA)	Sigma Aldrich	Cat#A3912
Fetal bovine Serum (FBS)	Life Technologies	Cat#A5256801
MES Running buffer solution	Sigma Aldrich	Cat# NP0002-02
Hoechst staining reagent	Invitrogen	Cat#H3570
Rapamycin	Sigma Aldrich	Cat#R8781
Low pH epitope retrieval solution	Agilent technologies	Cat#K8005
iBlots	Invitrogen	Cat#IB23002
G418	Sigma Aldrich	Cat#04727878001

**Critical commercial assays**		

Pierce BCA protein concentration	Pierce	Cat#23227
Mouse-on-mouse kit	Vector Laboratories	Cat#BMK2202

**Experimental models: Cell lines**		

Huh-7 Hepatocyte cell line	Lab of Prof. Loannis Spyrou. Linköping University	RRID: CVCL_0336

**Experimental models: Organisms/strains**		

(Thy-1)-h(A30P) alpha-synuclein	Laboratory of Professor Philip Khale	RRID: MGI:2652214

**Recombinant DNA**		

Cx32-mCherry	Addgene	Addgene Cat#55022
Alpha-synuclein-GFP	This paper	N/A

**Software and algorithms**		

Prism GraphPad version 10	Prism GraphPad	Graphpad https://www.graphpad.com/scientific-software/prism/
ImageJ (Fiji)		https://imagej.net/ij/

**Other**		

PT LINK Unit	Agilent, DAKO	Cat#PT200

### Experimental model and study participant details

#### Mammalian cell line

Huh-7 cells were obtained from Prof. Loannis Spyrou at Linköping University. Prior to use, cells were tested for mycoplasma using qPCR analysis (Eurofins Genomics).

#### Transgenic mouse and wild type line

The (Thy-1)-h(A30P) alpha-synuclein line was obtained from the laboratory of Prof. Martin Ingelsson at the Department of Public Health and Caring Sciences, Molecular Geriatrics, Rudbeck Laboratory, Uppsala University, Uppsala Sweden and Prof. Philip Khale from and the Laboratory of Functional Neurogenetics, Department of Neurodegeneration, Hertie Institute for Clinical Brain Research and German Center for Neurodegenerative Diseases, University of Tübingen, Tübingen, Germany. The transgenic ((Thy-1)-h(A30P)) line expressing human homozygous α-syn under control of the Thy-1 promoter on a CB57BL/6J background and their wild type counterpart,^25^ were housed in open cages on a 12:12 h dark: light cycle in a temperature- and humidity-controlled room and properly cared for by the pathogen free animal facility. All experiments involving mice were approved by the Local Animal Ethics Committee at Uppsala University. The use and care of the animals were conducted in accordance with the EU Directive 2010/63/EU for animal experiments.

### Method details

#### Generation of human α-syn assemblies

Recombinant α-syn was solubilized in PBS to generate monomers at a concentration of 5.0 mg/mL. To generate α-syn protein oligomers or fibrils (WT or 1–120 truncated form), we incubated α-syn monomers at 37°C for 5 days in an Eppendorf SS mini-shaker (Eppendorf) with constant shaking at either 350 or 1000 RPM, respectively.^49^ Generated protein assemblies were then aliquoted and stored at −80°C for further analysis.

#### Transmission electron microscopy (TEM) characterization of α-syn assemblies

The human α-syn oligomers and fibrillar assemblies were prepared for TEM analysis, as previously reported.^49^^,^^50^ Briefly, each sample was placed on carbon-coated 200-mesh grids, negatively stained with 1% uranyl acetate and analyzed with a Jeol JEM1230 (Jeol). Images were taken using a Gatan Orius CCD camera (Gatan).

#### Generation of Huh-Cx32 and huh-7-Cx32-α-syn human hepatocyte cell lines

To express Cx32 in HuH-7 cells,^75^ and generate stable cell lines, we transfected cells with plasmid vectors expressing human Cx32-mCherry (a gift from Michael Davidson, Addgene plasmid # 55022) according to the manufacturer’s instructions (Amaxa Nucleofector, Lonza).^10^ Transfected cells were then selected with appropriate antibiotics (G418, Sigma-Aldrich) followed by single-cell sorting using FACS into 96-well plates to generate clonal cell lines. To generate Huh-7-Cx32-α-syn cell lines, we transfected α-*syn*-GFP to the Huh-7-Cx32 cell line and clonal cell lines were then selected by FACS-based single cell sorting.

#### Hepatocyte α-synuclein treatment

HuH-7 human hepatocytes stably expressing Cx32 or Cx32-α-*syn*-GFP were treated with increasing concentrations of monomers, oligomers or sonicated fibrillar assemblies of α-syn (1–5 μM) in serum free MEM medium (with 1% glutamine and 1% penicillin/streptomycin) for different time points. Following α-syn treatment, cells were washed three times in PBS for 5 min and then either incubated with 4% PFA for ICC or incubated with TrypLE Express (Gibco) for 3–5 min at room temperature. Cells were then collected and pelleted by centrifugation at 13,000 RPM (Eppendorf) then lysed in RIPA buffer followed by a brief sonication to completely lyse cell pellets and determine protein concentration using the DC Assay (Bio-Rad).

#### Western blot analysis

All samples were resuspended to a final concentration of 1X Laemmli’s sample buffer, boiled for 5 min, separated on 4–20% SDS-PAGE gels (Sigma Aldrich) and transferred to nitrocellulose membranes using iBLOT kits (Life Technologies). Nonspecific protein binding was blocked by incubating membranes with 2% nonfat dry milk, followed by incubation with primary antibodies at 4°C overnight (Figure 1; Table 1). After rinsing membranes using Tween-tris-buffered saline (Medicago), we incubated them with peroxidase-conjugated species-appropriate secondary antibodies (Dako) for 1 h at RT, followed by ECL substrate (Bio-Rad) to visualize signal on a ChemiDoc XRS+ (Bio-Rad). Densitometric analysis was performed using ImageJ (Fiji), and values with arbitrary units were normalized to the signals obtained from total protein measured with loading controls (β-actin).

#### Liver tissue isolation

Male mice used for this study were anesthetized with isoflurane before being transcardially perfused with 0.9% saline. After perfusion, the livers were isolated and fixed in 4% PFA, followed by treatment with 70% ethanol before the tissue was paraffin embedded. Samples from 18-month male homozygous (Thy-1)-h[A30P] α-syn Tg (*n=7*) and wild type (WT) mice (*n=12*) were isolated.^25^

#### Immunohistochemistry

Liver tissue sections were processed for immunohistochemistry as previously reported^10^ and labeled with human α-syn antibodies, as detailed in Figure 1 and Table 1. Briefly, Paraffin-embedded liver tissue sections were rehydrated in Xylene (Sigma-Aldrich) followed by incubation in decreasing concentrations of ethanol (100, 95, 70 and 50%) and then rinsed in water for 10 min. Epitope retrieval was performed using acidic conditions according to the manufacturer’s instructions (Dako). Tissue sections were rinsed three times (5 min) in PBS, then incubated with 0.04% Triton X-100 in PBS for 1 h and processed for immunohistochemistry as described above, followed by Sudan Black staining to eliminate lipofuscin autofluorescence as previously reported.^63^^,^^64^^,^^65^

#### Immunocytochemistry

HuH-7-Cx32 or HuH-7-Cx32-α-syn hepatocytes were processed for immunohistochemistry (ICC) as previously reported.^50^ Briefly, cells fixed with 4% PFA were washed three times with PBS and permeabilized by incubation in 0.1% Triton X-100 in PBS for 20 min on ice. Next, cells were washed with PBS and incubated in 5% BSA for 1 h at RT, followed by incubation with primary antibodies overnight at 4°C (Table 1). The next day, cells were washed with PBS and incubated with respective fluorescently labeled secondary antibodies for 1 h (goat anti-mouse or goat anti-rabbit IgG Alexa Fluor 488, or 564, Life Technologies).

#### Confocal image analyses

To visualize fluorescence immunostaining, we used a Zeiss LSM 700 confocal microscope equipped with diode lasers (405-, 488-, or 555-lasers) used for excitation to acquire Z-stacks of consecutive confocal images.

#### Statistical analyses

Statistical analyses were performed using a two-way ANOVA with Tukey’s test when comparing multiple samples. For Western blot analyses, band intensities were quantified using ImageJ software (Fiji) software, and values with arbitrary units were normalized to the signal obtained from the protein loading control. In each data group, the results are expressed as the mean ± SEM. All data analyses were performed with GraphPad Prism 10.0 (La Jolla, CA). Findings were regarded as significant when ∗*p* < 0.05, ∗∗*p* < 0.01, ∗∗∗*p* < 0.001, ∗∗∗∗*p* < 0.0001.

#### Study approval

All procedures were performed according to the NIH Guide for the Care and Use of Experimental Animals and approved by Uppsala University in accordance with the EU Directive 2010/63/EU for animal experiments.

### Quantification and statistical analysis

Data was analyzed using one-way ANOVA with Tukey’s multiple comparison test. Data are represented as SEM and statistical significance as; ∗*p* = 0.05, ∗∗*p* = 0.01, ∗∗∗*p* = 0.001, and ∗∗∗∗*p* = 0.0001, respectively.
