# Supplementary material for: Accumulation of alpha-synuclein pathology in the liver exhibits post-translational modifications associated with Parkinson’s disease
Source: iScience. 2024 Nov 23;27(12):111448. doi: 10.1016/j.isci.2024.111448 (PMC11667178; doi:10.1016/j.isci.2024.111448)
Supplement: Document S1. Figures S1–S4 [file mmc1.pdf]

iScience, Volume *27*

Supplemental information

Accumulation of alpha-synuclein pathology in the liver exhibits post-translational modifications associated with Parkinson's disease

Martin Hallbeck, Sara Ekmark-Lewén, Philipp J. Kahle, Martin Ingelsson, and Juan F. Reyes

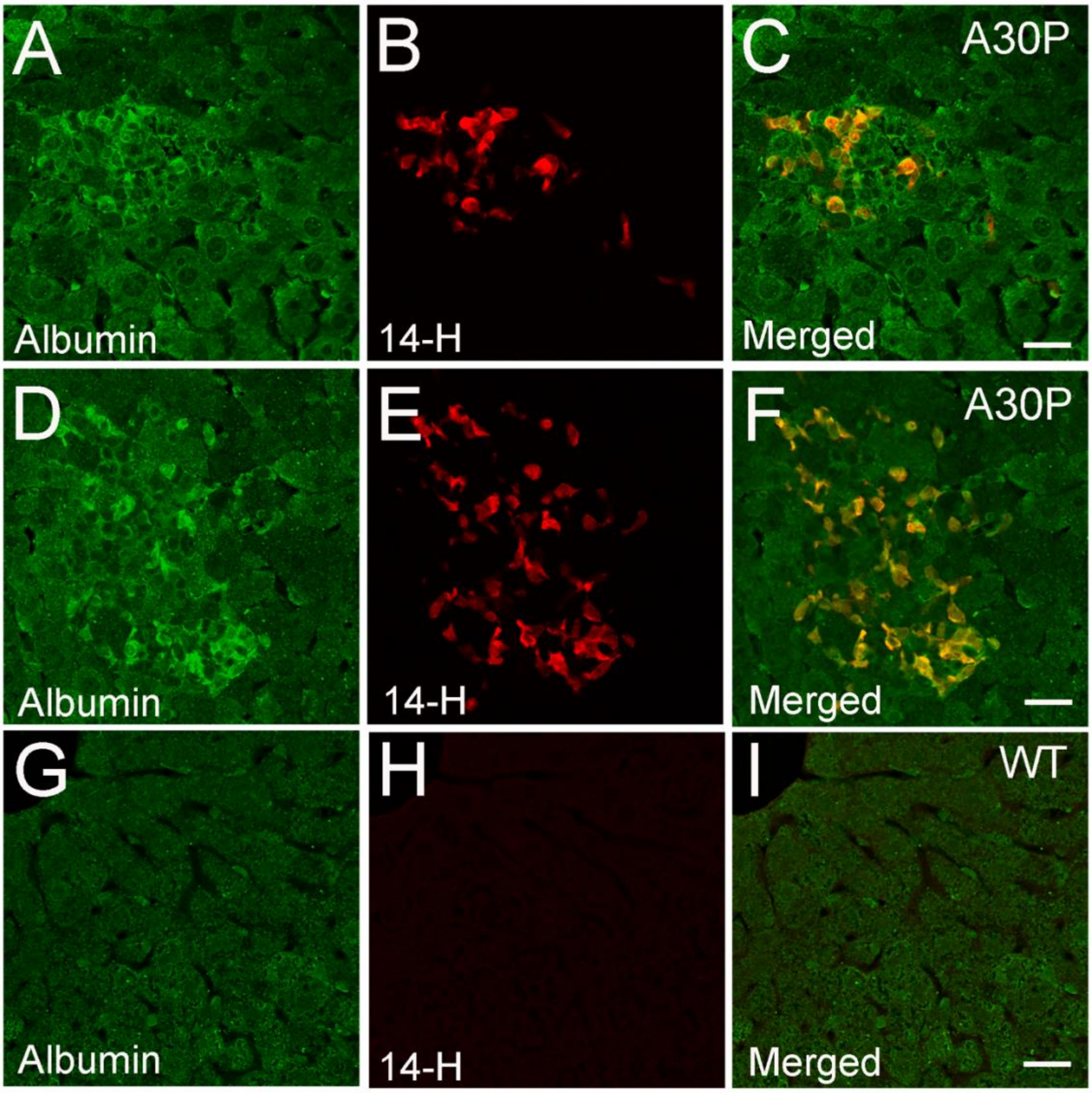

Figure S1. Human α-syn pathology in the liver localizes to hepatocellular structures. A-F) Tg (Thy-1)-h[A30P] and WT (G-I) mouse liver tissue sections immunolabeled with antibodies targeting albumin and 14-H to visualize α-syn pathology in the liver. Note the clear co-localization between α-syn and the hepatocellular structures. Bar = 25 μm

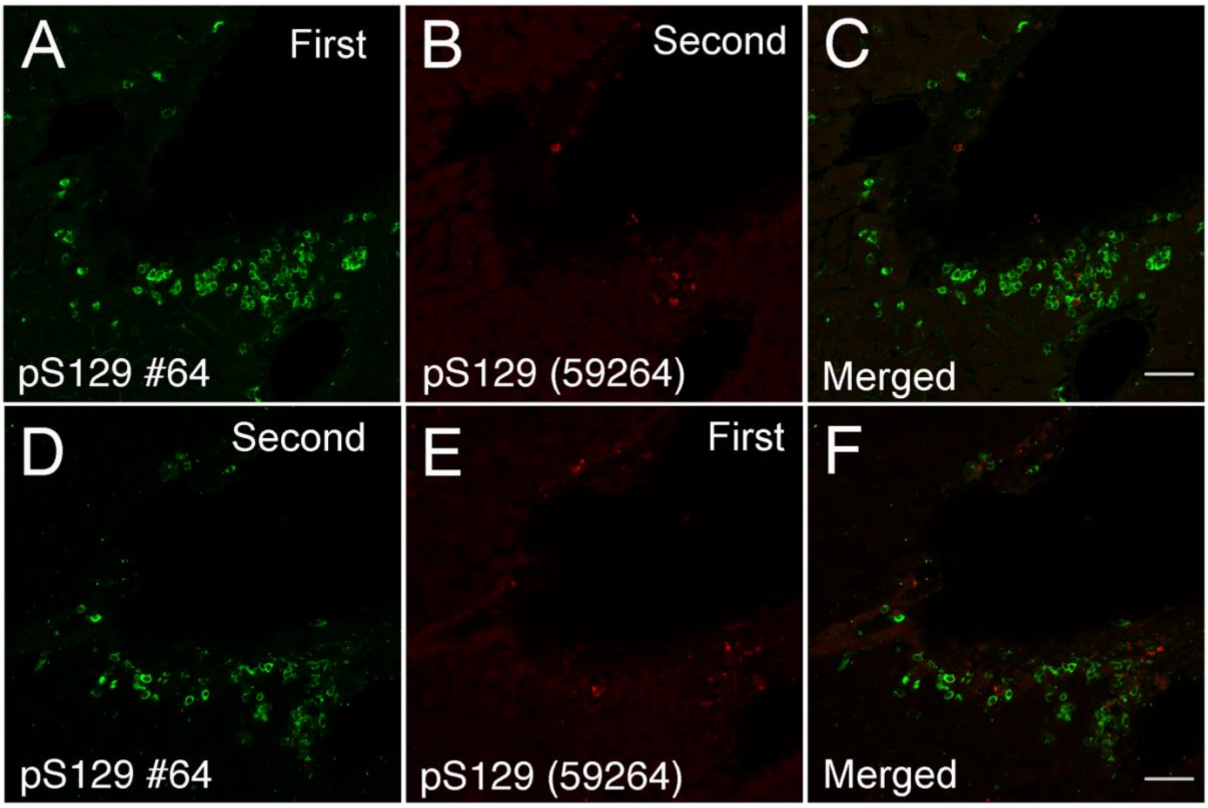

Figure S2. Phosphorylation at serine 129 (pS129) prevents 14-H binding. A-C) Mouse liver tissue sections labeled with pS129 (#64, first) for 24 hrs followed by 14-H for the same time period. D-F) Adjacent tissue sections labeled first with 14-H followed by pS129 (#64). Note the lack of co-localization between the two antibodies regardless of which antibody is added first. Bar = 50 µm

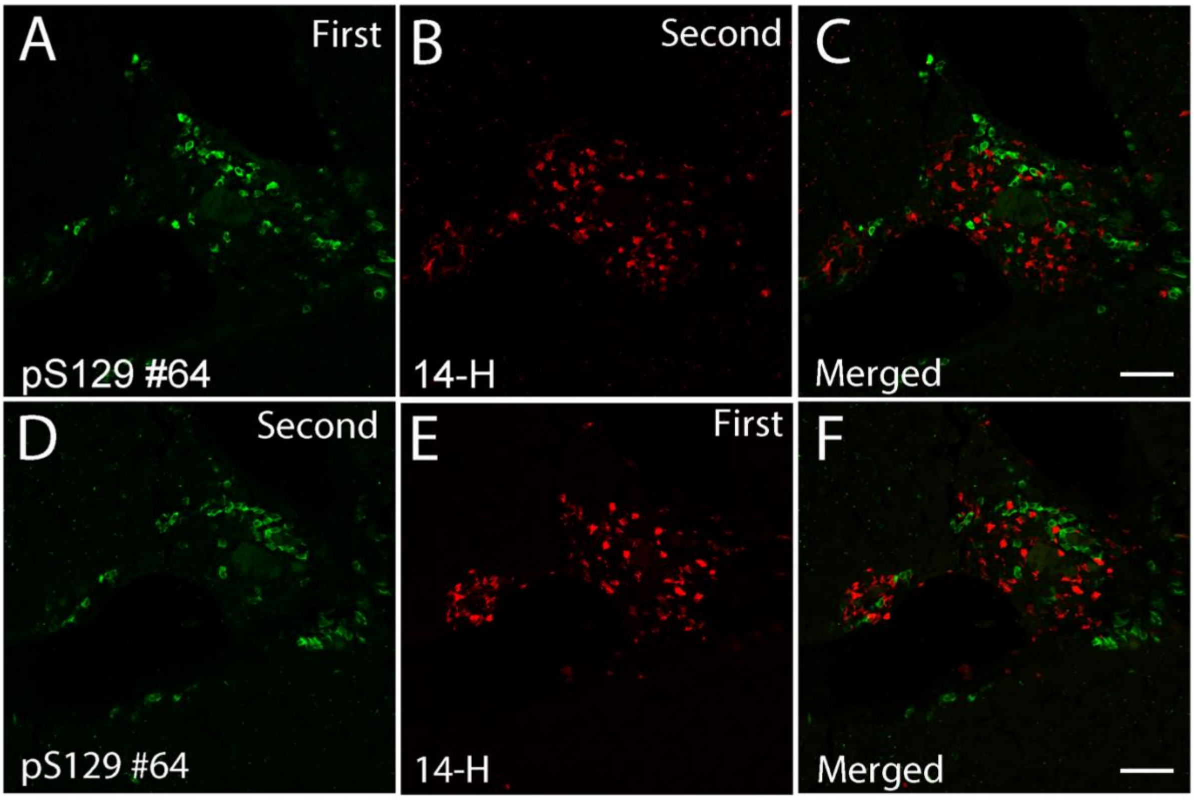

Figure S3. Dual pS129 antibody treatment does not affect α-syn reactivity in the (Thy-1)-h[A30P] liver A-C) Mouse liver tissue sections immunolabeled with pS129 (#64, first) for 24 hrs followed by pS129 (59264) for the same time period show no co-localization. D-F) Adjacent tissue sections labeled first with pS129 (59264) followed by pS129 (#64). Note the lack of co-localization between the two antibodies regardless of which antibody was added first. Bar = 50 μm

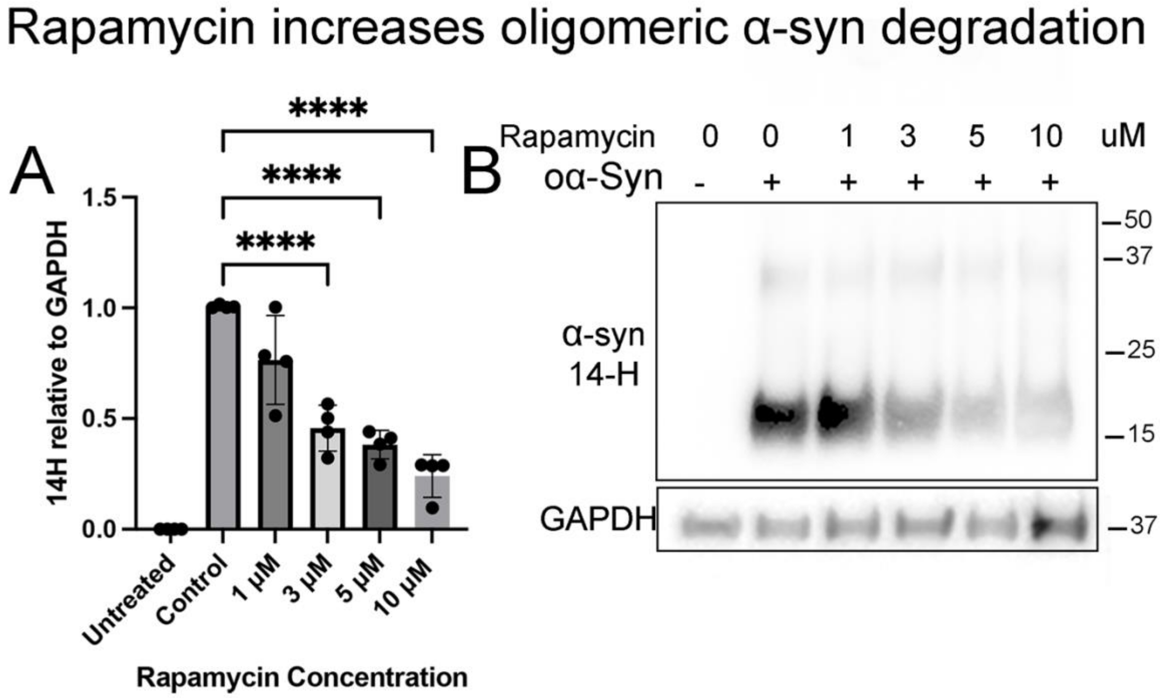

Figure S4. Rapamycin treatment increases α-syn oligomer degradation in a concentration dependent manner. A-B) Western blot analysis of Huh-7-Cx32 cells treated with different concentrations of Rapamycin in the presence of oα-syn assemblies blotted with the 14-H α-syn antibody and GAPDH was used as a loading control. Data was analyzed using one-way ANOVA with Tukey's multiple comparison test. Data are represented as SEM (*n=4, p=0.0001*).
